# *Acmella oleracea* and *Boswellia serrata* for Symptom Relief and Reduced Analgesic Use in Women with Dysmenorrhea

**DOI:** 10.1089/whr.2025.0056

**Published:** 2025-06-30

**Authors:** Maria Teresa Schettino, Maria Giovanna Vastarella, Gaetano Riemma, Ernesta Dores, Fabio Turco, Pasquale De Franciscis

**Affiliations:** ^1^ Department of Women, Child and General and Specialized Surgery, University of Campania “Luigi Vanvitelli”, Naples, Italy.; ^2^ Department of Acceptance, Emergency Room and Emergency Medicine, San Carlo Hospital, Potenza, Italy.; ^3^ Cannabiscientia SA, Lugano, Switzerland.

**Keywords:** anti-inflammatory, complementary therapy, herbal extract, pain management, NSAIDs reduction, symptom relief

## Abstract

**Background and Aim:**

Dysmenorrhea causes intense menstrual pain and symptoms such as cramps, headaches, and nausea. Nonsteroidal anti-inflammatory drugs (NSAIDs) offer relief but have limited efficacy and side effects, prompting interest in alternative therapies. This study evaluated a food supplement with *Acmella oleracea* and *Boswellia serrata* for dysmenorrhea symptom management in women with inadequate NSAIDs response.

**Methods:**

This single-center retrospective study included 33 women aged 20–35 with dysmenorrhea-related symptoms who had experienced insufficient relief from NSAIDs alone. Participants received the food supplement in addition to NSAIDs over three menstrual cycles. The primary outcome was the reduction in pain intensity. Secondary outcomes included changes in NSAIDs use and symptom prevalence (*e.g.,* cramps, lower back pain, and dyspareunia). Data were collected at baseline and after 3 months of treatment.

**Results:**

After 3 months, general pain and migraine decreased significantly, with mean Numeric Rating Scale scores decreasing from 8.2 (±1.02) to 6.8 (±1.08; *p* < 0.0001) and from 8.3 (±0.90) to 4.8 (±0.87; *p* < 0.0001), respectively. NSAIDs use decreased by 40%, with 46.2% of patients no longer requiring NSAIDs. Symptom-specific reductions included cramps, lower back pain, muscle tension, lower abdominal cramps, and nausea. Dyspareunia showed a 25% reduction. The supplement was well-tolerated, with no adverse events reported.

**Conclusions:**

These preliminary findings suggest that this food supplement may effectively reduce dysmenorrhea symptoms and reliance on NSAIDs in patients with insufficient response to conventional therapies. Further validation through randomized controlled trials is needed to confirm these results and establish the supplement’s role in dysmenorrhea management.

## Introduction

Dysmenorrhea, from the Greek *dys-* (difficulty), *emmēna* (menstruation), and *rhoíā* (flow), is one of the most common gynecological conditions, affecting 45% to 95% of women of reproductive age worldwide.^1^ Dysmenorrhea is characterized by lower abdominal or pelvic pain that begins with menstruation and typically lasts 48–72 hours. This pain is often accompanied by symptoms such as nausea, vomiting, fatigue, diarrhea, and, occasionally, fever.^2^

Dysmenorrhea is classified as mild, moderate, or severe based on pain intensity, impact on school or work activities, and the extent of analgesic use required for relief.^3,4^ Mild dysmenorrhea requires minimal analgesic intervention, moderate dysmenorrhea necessitates regular analgesic use, and severe dysmenorrhea can be debilitating, with conventional analgesics often providing limited relief, and may present with additional symptoms such as nausea and vomiting.

Previously dysmenorrhea was often attributed only to emotional or psychosocial factors. Recent clinical and experimental studies have identified complex physiological mechanisms.^5,6^ These include structural and connectivity alterations in brain regions involved in pain modulation, genetic factors, and heightened systemic inflammation, evidenced by elevated serum levels of nitric oxide and malondialdehyde, a marker of oxidative stress.^7–10^ Additionally, low levels of homocysteine and vitamin D have been implicated in increased pain sensitivity among affected women.^11,12^ Moreover, an imbalance in the levels of prostaglandins, vasopressin, and other mediators is associated with dysmenorrhea symptoms.^13,14^

The standard management of dysmenorrhea includes nonsteroidal anti-inflammatory drugs (NSAIDs), which inhibit prostaglandin synthesis, and hormonal contraceptives to suppress ovulation and reduce endometrial proliferation.^4,6,15^ While effective, these treatments are not suitable for all women and can carry adverse effects, including gastrointestinal irritation and, with hormonal therapies, an increased risk of cardiovascular events.^16,17^ Consequently, there is growing interest in complementary interventions, particularly food supplements with analgesic and anti-inflammatory properties.

Among these, *Acmella oleracea* and *Boswellia serrata* have shown potential in managing pain associated with dysmenorrhea. Acmella contains amino acids, triterpenoid acids, and alkylamines, which together provide anti-inflammatory and pain-relieving effects.^18^ In particular, the plant contains the bioactive compound spilanthol, which has demonstrated both anti-inflammatory and anesthetic effects by modulating pathways involved in pain perception.^19^ These properties suggest its potential utility in treating dysmenorrhea-related pain by modulating inflammatory pathways.^20^ Boswellia contains boswellic acids, such as the acetyl keto-beta-boswellic acid (AKBA), which inhibit 5-lipoxygenase, a key enzyme in the leukotriene pathway associated with inflammation, and thus induce analgesic relief.^21^ In a study with 60 women, an extract containing *B. serrata* exhibited remarkable menstrual pain relief as compared to the placebo.^22^ Recently, a fixed-dose combination of *A. oleracea* and *B. serrata* extracts that shows a synergistic effect has been developed through an isobolographic analysis and exerts an antiallodynic effect in a preclinical model of neuropathic pain.^23^

The present study evaluated the effects of a supplement containing both *A. oleracea* and *B. serrata* in women with primary or secondary dysmenorrhea who have not achieved adequate symptom control with NSAIDs alone. The primary aim was to assess improvements in dysmenorrhea-related pain, measured by changes in pain scores. Secondary outcomes included reductions in associated symptoms such as migraine, NSAIDs intake, abdominal pain, and sexual health impairments.

## Methods

### Study design

This study was conducted as a retrospective, observational study aimed at evaluating the effectiveness of a food supplement containing *A. oleracea* and *B. serrata* extracts (Nervana®, Sanitas Farmaceutici, Italy) in managing symptoms associated with dysmenorrhea. The study was performed in accordance with the STROBE guidelines for observational studies.^24^ The study took place at the Vanvitelli University Hospital, a tertiary care center with specialized gynecology and obstetrics units. Recruitment occurred between January and August 2024, focusing on patients visiting the gynecology and obstetrics units for dysmenorrhea-related complaints. The study was approved by the “Comitato Etico Territoriale Campania 2” ethic committee, prot.002933/I 01/10/2024. All patients signed an informed consent form prior to participation. All procedures adhere to ethical standards set by the responsible committee on human experimentation (both institutional and national), as well as the Helsinki Declaration of 1975, as revised in 2008.

### Participants

This study focused on a population of women with mild, moderate, or severe dysmenorrhea. Enrollment involved a screening process for patients presenting with dysmenorrhea-related issues at the gynecology and obstetrics units. The diagnosis of dysmenorrhea was confirmed after a comprehensive physical examination conducted at the first visit (baseline), which included a gynecological examination, transvaginal ultrasound, and cervical screening tests. Complete details of the physical examination are available upon request. Patients who met the inclusion criteria were subsequently enrolled in the study.

*Inclusion criteria:*

- Age: 20–35 years;
- Diagnosis of dysmenorrhea with associated symptoms such as headache, joint pain, mood disturbances, or sexual dysfunction;
- A baseline pain score of >7 on the Numeric Rating Scale (NRS);
- Inadequate response to NSAIDs for pain relief.

*Exclusion criteria:*

- Pregnancy or lactation;
- Concurrent use of other supplements;
- Age outside the 20–35 years range;
- Coexisting medical conditions;
- History of cancer;
- Inability to provide informed consent.

### Intervention

The food supplement containing natural extracts of *A. oleracea* and *B. serrata* was administered as an adjunct to the standard therapeutic protocol for dysmenorrhea, which included NSAID treatment during the menstrual cycle. Patients selected for the study had previously shown unsatisfactory responses to NSAID therapy alone. Each participant took the food supplement at a dosage of two tablets per day, beginning 7 days prior to the menstrual cycle and continuing until the end of menstruation, over the course of three consecutive cycles. The follow-up period was 3 months after the starting of the treatment.

### Data collection

Data were collected at:

- Baseline: Patient diagnosis, clinical history, and initial NSAID therapy were recorded.
- Visit 1 (3 months after the food supplement initiation): Evaluation of pain and associated dysmenorrhea symptom changes.

### Clinical investigation endpoints

#### Primary endpoint

The primary endpoint of this study was the reduction in dysmenorrhea-associated pain, as measured by the NRS, after 3 months of treatment with the food supplement.

#### Secondary endpoints

- Decrease in NSAIDs usage required for managing dysmenorrhea-associated pain over the course of the study;
- Improvements in migraine related to dysmenorrhea;
- Evaluation of changes in symptoms associated with dysmenorrhea, including sexual function, muscle pain, and others, following three months of the food supplement treatment.

### Numeric rating scale (NRS)

The NRS is a widely used questionnaire for assessing pain intensity.^25^ In this study, the NRS was employed to quantify dysmenorrhea-related pain among participants. The scale ranges from 0 to 10, with “0” indicating no pain and “10” representing the worst imaginable pain. To ensure consistency, questionnaires were completed in a quiet setting, preferably in the same location each time, at a consistent point during the visit. No explicit or implied time limit was set, allowing participants to complete the questionnaire at their own pace.

### Food supplement

The food supplement chosen was Nervana®, a patented food supplement formulated with standardized extracts of *A. oleracea* and *B. serrata* . Nervana is presented in a gastro-protected formulation. *B. serrata* is integrated into a phytosome complex, enhancing its absorption *via* a phospholipidic structure. *A. oleracea* is combined with beta-cyclodextrin, a cyclic oligosaccharide that accelerates delivery into the body.

Each daily dose of two tablets provides:

- *B. serrata* (standardized to 25% triterpenic acids): 120 mg
- *A. oleracea* (standardized to 3% alkamides): 120 mg

### Statistical analysis

The sample size was calculated to achieve a power of 80% at a 5% significance level (α = 0.05), assuming a medium effect size (dz = 0.5) in the reduction of NRS scores after 3 months of *A. oleracea* and *B. serrata* treatment. Based on this calculation, a minimum of 33 patients was required. For the primary outcome (change in NRS scores), a two-tailed paired samples Student’s *t*-test was conducted, with a significance level set at 0.05. Mean differences are reported along with their standard deviations (SDs) and 95% confidence intervals (CIs) to reflect the variability in pain score reductions. For secondary binary outcomes, such as NSAID use before and after treatment, McNemar’s test was used to assess the significance of changes in paired categorical data. The absolute risk reduction was also reported. For other categorical symptoms (*e.g.,* cramps, nausea, dyspareunia), descriptive analysis was performed by calculating the absolute change in the number and percentage of patients reporting each symptom before and after treatment.

## Results

### Patients and symptoms at baseline

A total of 33 patients were enrolled in the study. The most frequently reported symptom at baseline was lower abdominal cramps, experienced by 20 patients (60.6%). Other common symptoms included migraine in 12 patients (36.4%), lower back pain in 8 patients (24.2%), muscle tension in 6 patients (18.1%), and diffuse muscle pain in 6 patients (18.1%). Additionally, four patients (12.1%) each experienced mastodynia (breast pain) and dyspareunia (pain during intercourse). Less frequent symptoms included nausea in two patients (6.0%) and fatigue, reported by one patient (3.0%). At baseline, all patients demonstrated an inadequate response to NSAIDs. The most used NSAIDs were ibuprofen and naproxen, followed by paracetamol. Baseline characteristics of the patients included in the study are presented in Table 1.

**Table 1.** Prevalence of Symptoms Experienced by Patients at Baseline, Prior to Treatment with the *Acmella oleracea* and *Boswellia serrata* Food Supplement

Symptoms at baseline	Number of patients	Percentage (%)
Lower abdominal cramps	20	60.6%
Migraine	12	36.4%
Lower back pain	8	24.2%
Muscle tension	6	18.1%
diffuse muscle pain	6	18.1%
Mastodynia	4	12.1%
Dyspareunia	4	12.1%
Nausea	2	6.0%
Fatigue	1	3.0%

Each symptom is listed with the number of patients who reported it and the corresponding percentage of the total study population. The symptoms include common dysmenorrhea-related complaints, such as abdominal cramps, headache, and lower back pain, and other associated symptoms such as muscle pain, breast pain, dyspareunia (pain during intercourse), and fatigue. The data provides an overview of the variety and frequency of symptoms within the study cohort at baseline.

### Effects of the supplement on general pain and migraine

**FIG. 1.**
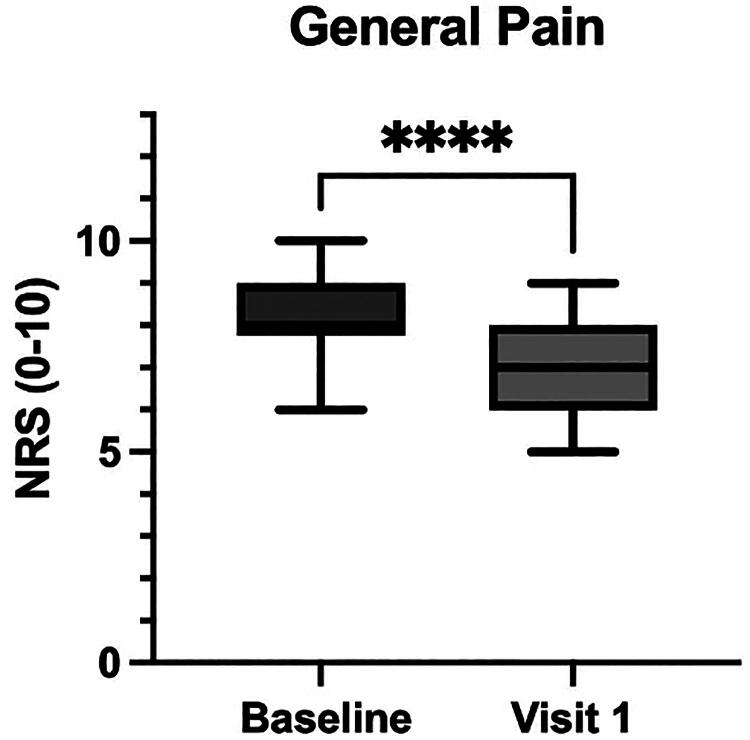
Reduction in General Pain Scores following treatment with the *Acmella oleracea* and *Boswellia serrata* extract. The graph illustrates the Numeric Rating Scale (NRS) scores for general pain at baseline and at visit 1 (after 3 months of treatment with the *Acmella oleracea* and *Boswellia serrata* extract). A significant reduction in pain levels was observed from baseline to visit 1, as indicated by the asterisks (****), with *p* < 0.0001.

**FIG. 2.**
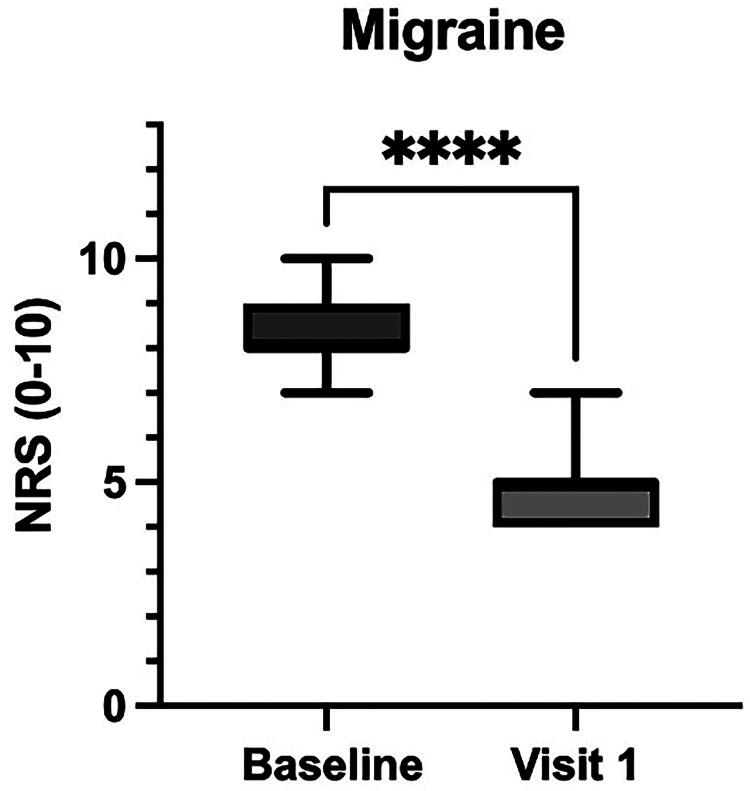
Reduction in migraine scores following treatment. With the *Acmella oleracea* and *Boswellia serrata* extract. The graph displays the Numeric Rating Scale (NRS) scores for migraine pain at baseline and at visit 1 (after 3 months of treatment with the *Acmella oleracea* and *Boswellia serrata* extract). A significant reduction in migraine severity was observed from baseline to Visit 1, as indicated by the asterisks (****), with *p* < 0.0001.

The primary outcome, general pain reduction, showed a statistically significant decrease from a baseline mean score of 8.2 (SD = 1.02) to a follow-up mean score of 6.8 (SD = 1.08), with a mean difference of 1.4 (95% CI: 1.0–1.8, *p* < 0.0001) (Fig. 1). This indicates an overall improvement in pain perception following the intervention. For patients reporting migraine symptoms associated with dysmenorrhea, the mean NRS migraine score decreased from 8.3 (SD = 0.90) to 4.8 (SD = 0.87), yielding a mean reduction of 3.5 (95% CI: 2.8–4.2; *p* < 0.0001) (Fig. 2).

### Effect of the supplement on NSAIDs usage

**FIG. 3.**
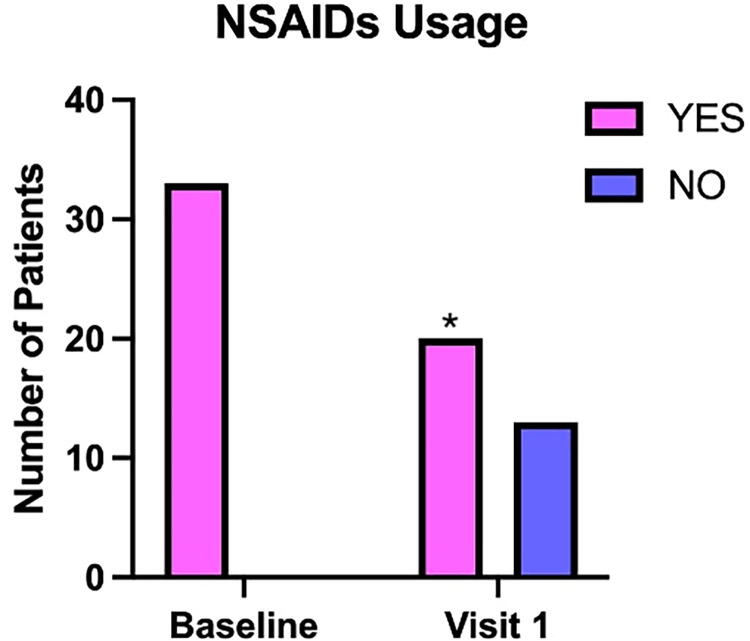
Reduction in NSAIDs usage following treatment with the *Acmella oleracea* and *Boswellia serrata* extract. The bar chart illustrates the number of patients who reported using NSAIDs at baseline and after 3 months of treatment with Nervana® (visit 1). “YES” (pink) indicates patients who used NSAIDs, and “NO” (blue) indicates those who did not. At baseline, all patients used NSAIDs for managing dysmenorrhea symptoms. After 3 months, there was a notable reduction in NSAID usage, with the proportion of NSAID users decreasing from 100% to 39.4%, corresponding to an absolute risk reduction of 60.6%. This reduction in NSAID usage was statistically significant, as assessed by McNemar’s test (*p = 0.0001).

At visit 1, following three months of treatment with the *A. oleracea* and *B. serrata* food supplement, there was a notable reduction in NSAIDs use among patients (Fig. 3). At baseline, all patients were using NSAIDs to manage dysmenorrhea symptoms, even if with an inadequate response. After 3 months of treatment, the number of patients using NSAIDs decreased from 33 (100%) at baseline to 13 (39.4%). This corresponds to an absolute risk reduction of 60.6%. McNemar’s test showed a statistically significant reduction in NSAID use (*p* = 0.0001).

### Effect of the supplement on additional dysmenorrhea symptoms

After 3 months of treatment with the *A. oleracea* and *B. serrata* food supplement, there was a notable reduction in the prevalence of various symptoms associated with dysmenorrhea (Table 2). The prevalence of abdominal cramps decreased by 20%. Migraine was reduced by 33.3%, and the prevalence of lower back pain decreased by 25%. Muscle tension and diffuse muscle pain each showed a 33.3% reduction in occurrence. Nausea demonstrated one of the most substantial improvements, with a 50% reduction. Additional symptoms, including breast pain and dyspareunia, each exhibited a 25% reduction following treatment. Fatigue remained unchanged post-treatment.

**Table 2.** Symptom Reduction at Visit 1 Following the Treatment with the *Acmella oleracea* and *Boswellia serrata* Food Supplement

Symptoms at visit 1	Reduction in symptom prevalence (percentage)
Lower abdominal cramps	20%
Migraine	33.3%
Lower back pain	25%
Muscle tension	33.3%
Diffuse muscle pain	33.3%
Breast pain	25%
Dyspareunia	25%
Nausea	50%
Fatigue	No change

This table summarizes the percentage of reduction in the prevalence of various symptoms associated with dysmenorrhea among patients, after 3 months of treatment with the *A. oleracea* and *B. serrata* food supplement. Each symptom is listed with the corresponding percentage of patients who reported decreased prevalence at visit.

### Safety

The treatment with the *A. oleracea* and *B. serrata* food supplement was well tolerated among all participants. Throughout the 3-month follow-up, no adverse events were reported by any of the patients.

## Discussion

This retrospective study assessed the ability of a food supplement containing *A. oleracea* and *B. serrata* to ameliorate dysmenorrhea-related symptoms in patients with an inadequate response to NSAIDs for pain relief. Our data show that over a 3-month period, patients reported reductions in general pain, migraines, cramps, and other associated symptoms, with no adverse events observed. These findings suggest that the *A. oleracea* and *B. serrata* food supplement could serve as a complementary approach for managing dysmenorrhea, particularly in patients seeking alternatives to conventional therapies such as NSAIDs.

The improvement in general pain and migraines observed aligns with the known anti-inflammatory and analgesic properties of *A. oleracea* and *B. serrata*.^18,21^The primary active compound in *A. oleracea*, spilanthol, has been found to exhibit analgesic effects by modulating the central pain pathway and reducing the release of pro-inflammatory mediators.^18,20^ In experiments with mice, the treatment with extracts from *A. oleracea* reduced both neuropathic and postoperative pain, inducing antinociceptive effects without causing adverse effects.^26,27^ Similarly, boswellic acids in *B. serrata*, especially AKBA, inhibit 5-lipoxygenase, a key enzyme in the inflammatory pathway associated with leukotriene synthesis.^21,22,28^ This inhibition may reduce inflammation and pain sensitivity, explaining the reductions observed in dysmenorrhea symptoms such as cramps and lower back pain. Recent preclinical studies suggest that the therapeutic effects of Acmella oleracea and Boswellia serrata may extend beyond peripheral anti-inflammatory mechanisms to include central modulation of spinal sensitization. It has been demonstrated that spinal hyperexcitability and microglial activation contribute to chronic pelvic pain in mouse models and that treatment with *A. oleracea* and *B. serrata* extracts reduced vulvar hypersensitivity, spinal microgliosis, and neuronal overactivity.^23,29^ Additionally, Infantino et al. (2025) emphasized the central role of spinal microglial activation in the maintenance of chronic pelvic pain and proposed it as a therapeutic target.^30^ These findings support the hypothesis that *A. oleracea* and *B. serrata* may alleviate dysmenorrhea symptoms through both peripheral and central mechanisms. In a randomized controlled trial (RCT) with 48 participants, a *B. serrata* extract exerted an anti‐inflammatory activity, improving patients’ physical and functional ability and reducing the pain.^31^ Another RCT found that, in patients with knee pain, *Boswellia* was safe and effective in reducing pain and improving the quality of life of patients.^32^ Further supporting this, the observed reduction in muscle tension, diffuse muscle pain, and other symptoms suggests that the *A. oleracea* and *B. serrata* food supplement may provide broader symptom relief beyond just general pain management.

The significant reduction in NSAIDs use among patients highlights the potential of the *A. oleracea* and *B. serrata* food supplement to serve as an alternative or complementary option for symptom management in dysmenorrhea. NSAIDs, while effective for short-term relief, are often associated with adverse effects, particularly gastrointestinal issues, which can limit long-term use.^16^ By reducing reliance on NSAIDs, the *A. oleracea* and *B. serrata* food supplement may offer patients a safer option with fewer side effects, making it suitable for those needing ongoing dysmenorrhea management. Moreover, the observed symptom-specific reductions, such as the decrease in lower abdominal cramps and nausea, further underscore the *A. oleracea* and *B. serrata* food supplement’s potential in addressing dysmenorrhea-related symptoms. An interesting area for further exploration is the potential reduction in dyspareunia, or pain during intercourse, which could positively impact sexual function in patients. Dyspareunia is often reported by individuals with dysmenorrhea, partly due to inflammation and muscle tension associated with menstrual pain.^33^ The compounds in the extract may reduce inflammatory responses and alleviate muscle tension, which are potential contributors to dyspareunia.

The compounds present in the extract may act synergistically to target various aspects of dysmenorrhea, thereby providing comprehensive relief. Indeed, our study and the existing literature support the potential analgesic and anti-inflammatory benefits of these natural compounds.

However, these findings are based on a retrospective analysis and should be interpreted with caution. The lack of randomization, control group, and reliance on self-reported measures introduce potential biases and limitations that restrict the ability to generalize these results. While the observed reductions in NSAIDs use and symptom relief are encouraging, further validation through large-scale RCTs is necessary to confirm the efficacy and safety of the *A. oleracea* and *B. serrata* food supplement for dysmenorrhea management. Such studies would also allow for more robust exploration of potential benefits in related areas, such as dyspareunia and sexual function. Until validated by RCTs, the findings from this study should be considered preliminary, and the *A. oleracea* and *B. serrata* food supplement should be viewed as a potential complementary approach rather than a definitive alternative to established therapies.

## Conclusion

This study offers preliminary evidence that the supplement containing *A. oleracea* and *B. serrata* extracts (Nervana) may be useful in managing dysmenorrhea-related symptoms, particularly in improving general pain and lowering reliance on NSAIDs. The observed improvements in specific symptoms, including migraine, cramps, and nausea, highlight the potential of the food supplement as a complementary option for patients seeking alternatives or adjuncts to conventional therapies. Given its favorable safety profile and promising results, further validation in larger RCTs is essential to confirm these findings and establish the positive results obtained with the food supplement for dysmenorrhea management.

## Data References

Raw data can be accessed upon request to the corresponding author.

## Abbreviations Used

AO: Acmella oleracea
BS: Boswellia serrata
CI: Confidence Interval
NRS: Numeric Rating Scale
NSAIDs: Nonsteroidal Anti-Inflammatory Drugs
OR: Odds Ratio
RCT: Randomized Controlled Trial
SD: Standard Deviation
STROBE: Strengthening the Reporting of Observational Studies in Epidemiology
